# Supplementary material for: Effect of Thidiazuron on Terpene Volatile Constituents and Terpenoid Biosynthesis Pathway Gene Expression of Shine Muscat (Vitis labrusca × V. vinifera) Grape Berries
Source: Molecules. 2020 Jun 2;25(11):2578. doi: 10.3390/molecules25112578 (PMC7321343; doi:10.3390/molecules25112578)
Supplement: Supplementary file 1 [file molecules-25-02578-s001.pdf]

Table S1. Quantitative standards and calibration curves for the quantification of free and bound terpene compounds in grape.

Compounds	Free compounds		Bound compounds	
	Calibration curves	r^2	Calibration curves	r^2
**Terpenes**				
Linalool	y = 2E+07x + 5E+08	0.9917	y = 2E+07x + 3E+08	0.9843
Hotrienol	y = 1E+06x + 2E+07	0.987	y = 1E+06x + 2E+07	0.9964
α-Terpineol	y = 6E+07x + 2E+08	0.9908	y = 2E+07x + 2E+07	0.9864
α-Citral	y = 2E+06x - 2E+06	0.9968	y = 143076x + 2E+06	0.9904
Citronellol	y = 2E+07x + 7E+06	0.9918	y = 5E+06x + 1E+07	0.9930
Neral	y =476659x + 3E+06	0.9871	y = 776229x + 869192	0.9964
Geraniol	y = 1E+06x + 5E+07	0.9616	y = 8E+06x - 3E+07	0.9975
Geranic acid	y = 5E+06x - 4E+06	0.9948	y = 1E+07x + 2E+08	0.9941
Citronellal	y = 2E+06x - 3E+06	0.9905	y = 5E+06x + 2E+06	0.9964
B-myrcene	y = 1E+06x + 1E+06	0.9944	y = 2E+06x - 2E+06	0.9989
D-limonene	y = 2E+06x - 716796	0.9981	y = 781898x + 952605	0.9982
Myrtenol	y = 7E+06x + 6E+07	0.9837	y = 5E+06x + 3E+07	0.9717
(*E*)-furanoid linalool	y = 9E+06x + 5E+07	0.988	y = 2E+07x + 1E+08	0.9961
(*Z*)-furanoid linalool	y = 3E+07x - 8E+07	0.9944	y = 4E+06x + 3E+07	0.9945

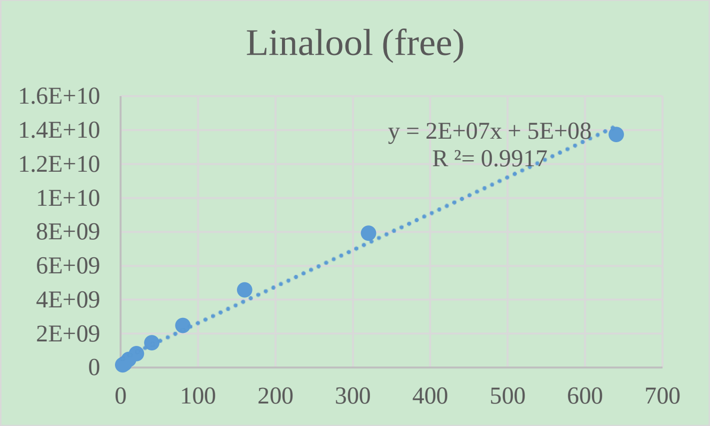
Linalool (free)
y = 2E+07x + 5E+08
R ²= 0.9917
1.6E+10
1.4E+10
1.2E+10
1E+10
8E+09
6E+09
4E+09
2E+09
0
0 100 200 300 400 500 600 700

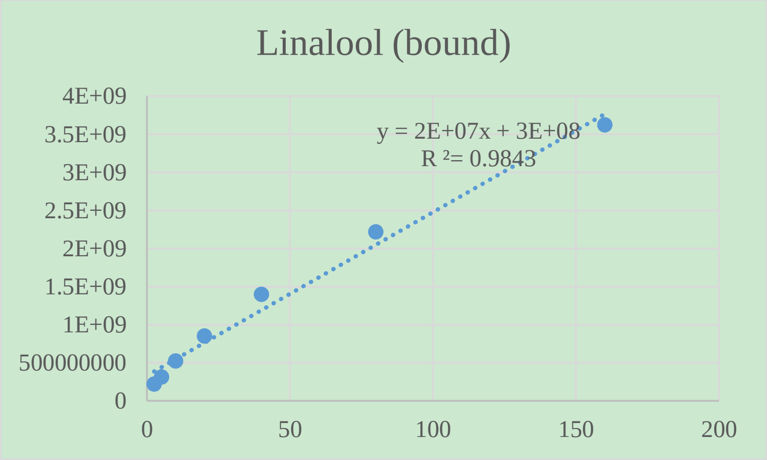
Linalool (bound)
y = 2E+07x + 3E+08
R ²= 0.9843
4E+09
3.5E+09
3E+09
2.5E+09
2E+09
1.5E+09
1E+09
500000000
0
0 50 100 150 200

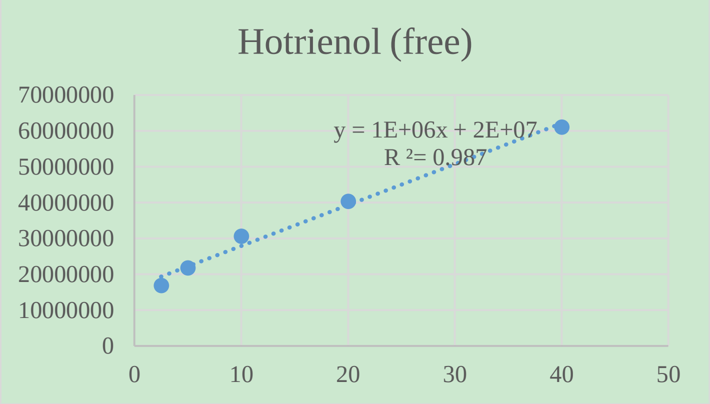
Hotrienol (free)
y = 1E+06x + 2E+07
R ²= 0.987
70000000
60000000
50000000
40000000
30000000
20000000
10000000
0
0 10 20 30 40 50

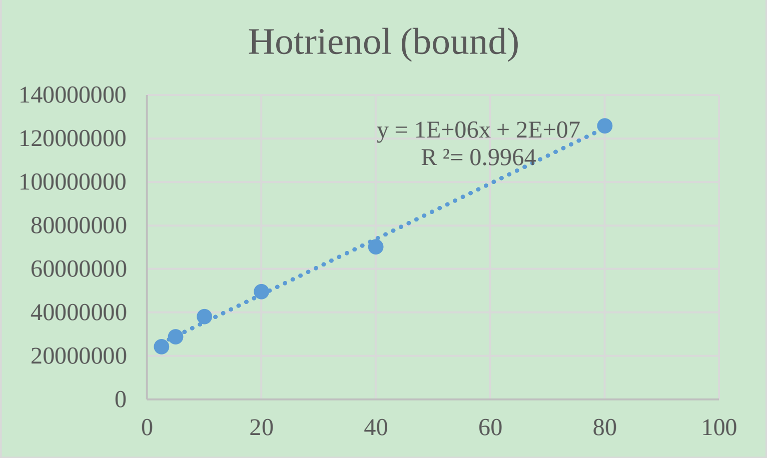
Hotrienol (bound)
y = 1E+06x + 2E+07
R ²= 0.9964
140000000
120000000
100000000
80000000
60000000
40000000
20000000
0
0 20 40 60 80 100

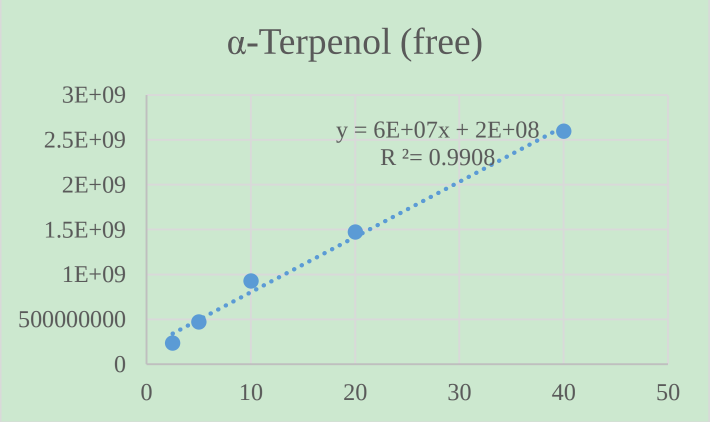
α-Terpenol (free)
y = 6E+07x + 2E+08
R ²= 0.9908
3E+09
2.5E+09
2E+09
1.5E+09
1E+09
500000000
0
0 10 20 30 40 50

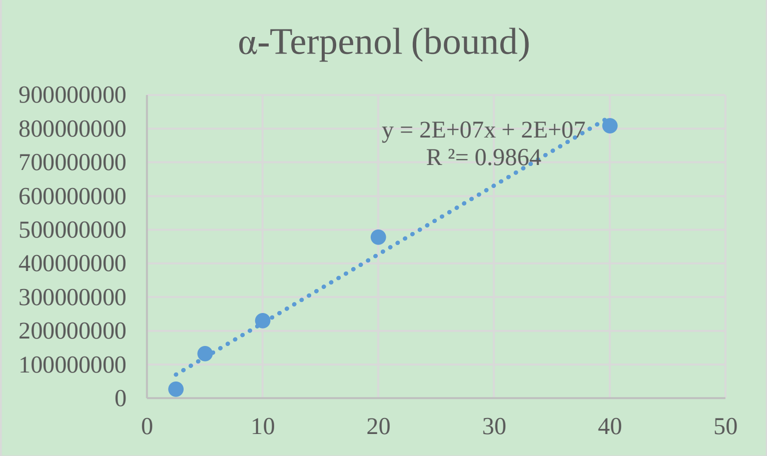
α-Terpenol (bound)
y = 2E+07x + 2E+07
R ²= 0.9864
900000000
800000000
700000000
600000000
500000000
400000000
300000000
200000000
100000000
0
0 10 20 30 40 50

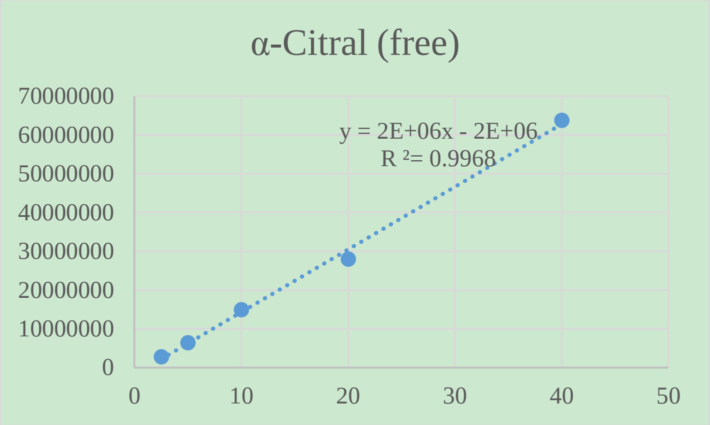
α-Citral (free)
y = 2E+06x - 2E+06
R ²= 0.9968
70000000
60000000
50000000
40000000
30000000
20000000
10000000
0
0 10 20 30 40 50

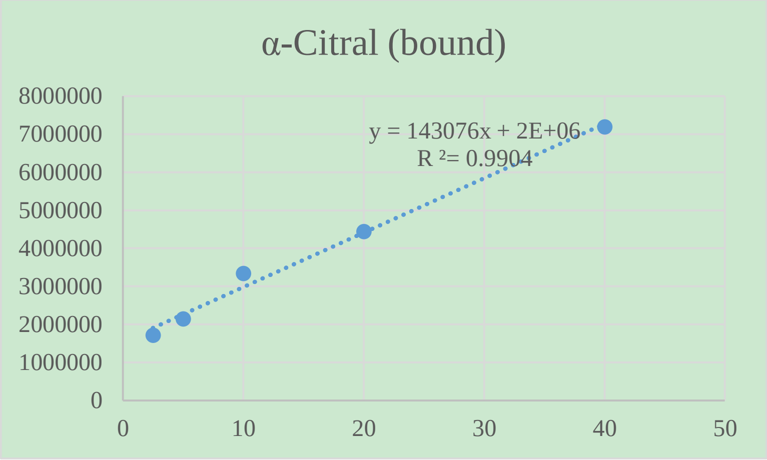
α-Citral (bound)
y = 143076x + 2E+06
R ²= 0.9904
8000000
7000000
6000000
5000000
4000000
3000000
2000000
1000000
0
0 10 20 30 40 50

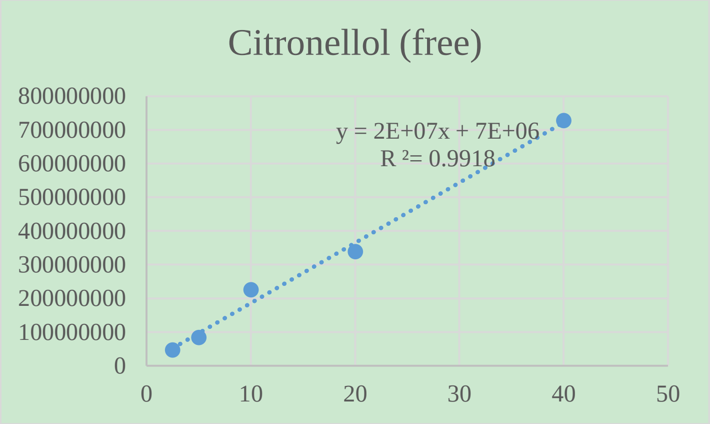
Citronellol (free)
y = 2E+07x + 7E+06
R ²= 0.9918
800000000
700000000
600000000
500000000
400000000
300000000
200000000
100000000
0
0 10 20 30 40 50

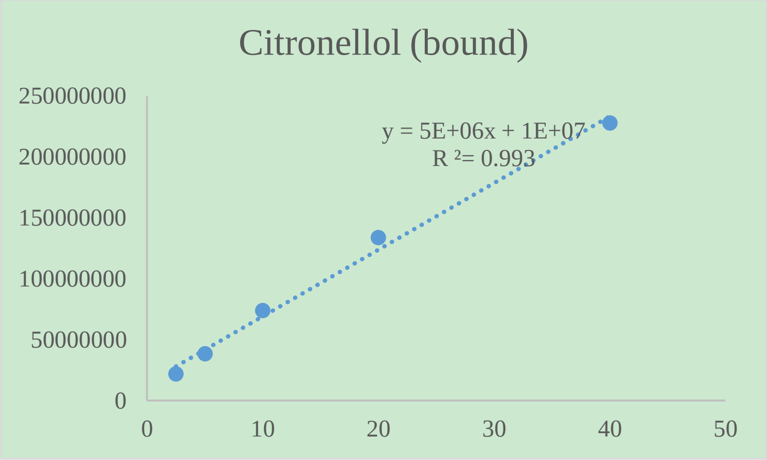
Citronellol (bound)
y = 5E+06x + 1E+07
R ²= 0.993
250000000
200000000
150000000
100000000
50000000
0
0 10 20 30 40 50

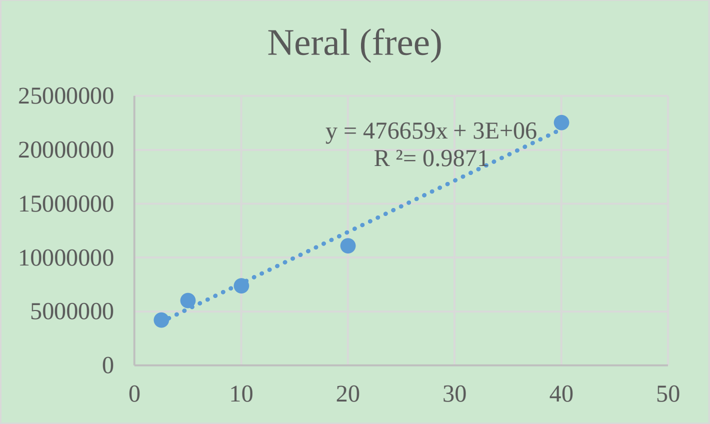
Neral (free)
y = 476659x + 3E+06
R ²= 0.9871
25000000
20000000
15000000
10000000
5000000
0
0 10 20 30 40 50

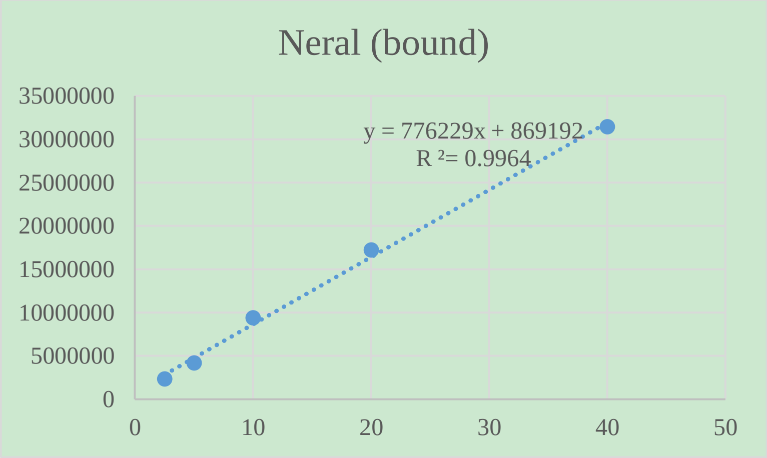
Neral (bound)
y = 776229x + 869192
R ²= 0.9964
35000000
30000000
25000000
20000000
15000000
10000000
5000000
0
0 10 20 30 40 50

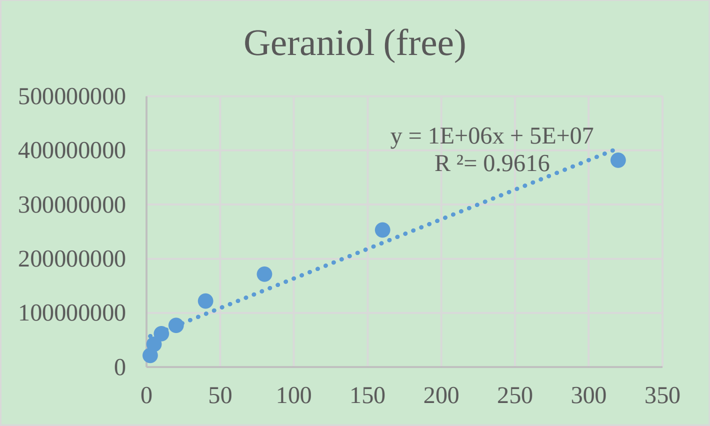
Geraniol (free)
y = 1E+06x + 5E+07
R ²= 0.9616
500000000
400000000
300000000
200000000
100000000
0
0 50 100 150 200 250 300 350

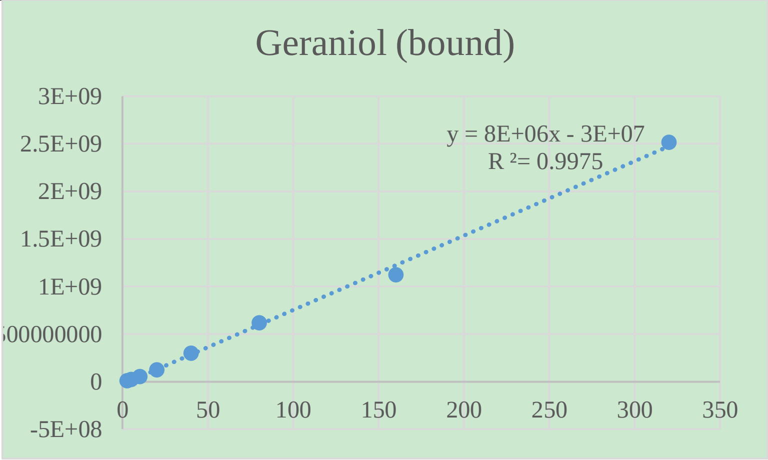
Geraniol (bound)
y = 8E+06x - 3E+07
R ²= 0.9975
3E+09
2.5E+09
2E+09
1.5E+09
1E+09
0
-5E+08
0 50 100 150 200 250 300 350

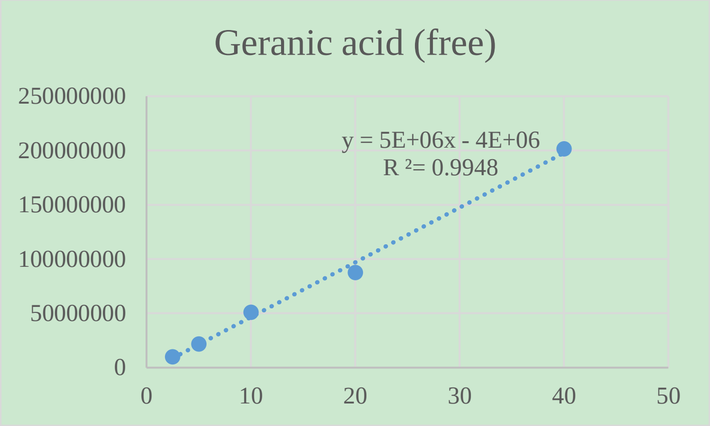
Geranic acid (free)
y = 5E+06x - 4E+06
R ²= 0.9948
250000000
200000000
150000000
100000000
50000000
0
0
10
20
30
40
50

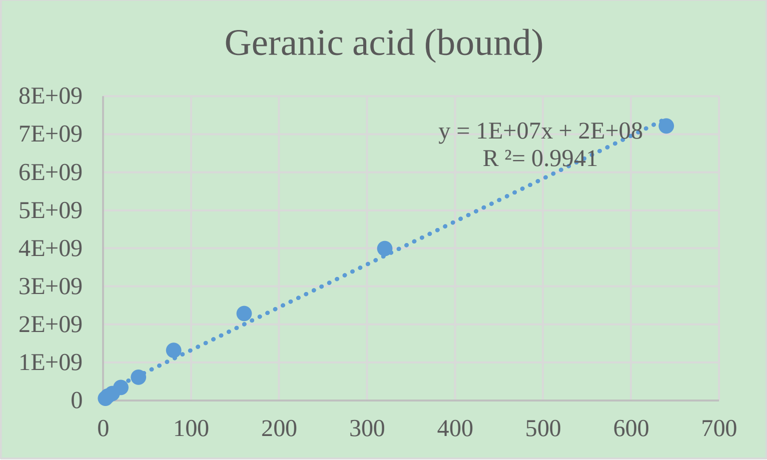
Geranic acid (bound)
y = 1E+07x + 2E+08
R ²= 0.9941
8E+09
7E+09
6E+09
5E+09
4E+09
3E+09
2E+09
1E+09
0
0
100
200
300
400
500
600
700

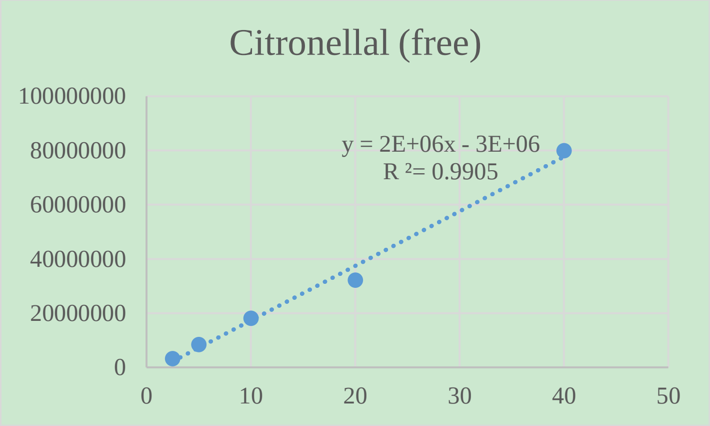
Citronellal (free)
y = 2E+06x - 3E+06
R ²= 0.9905
100000000
80000000
60000000
40000000
20000000
0
0
10
20
30
40
50

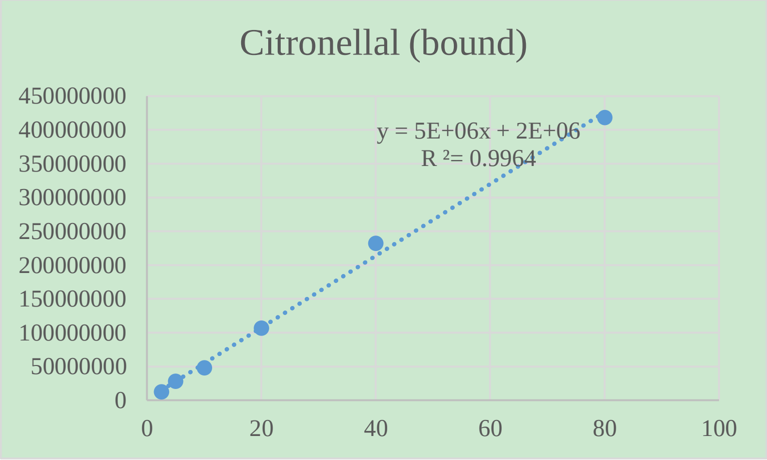
Citronellal (bound)
y = 5E+06x + 2E+06
R ²= 0.9964
450000000
400000000
350000000
300000000
250000000
200000000
150000000
100000000
50000000
0
0
20
40
60
80
100

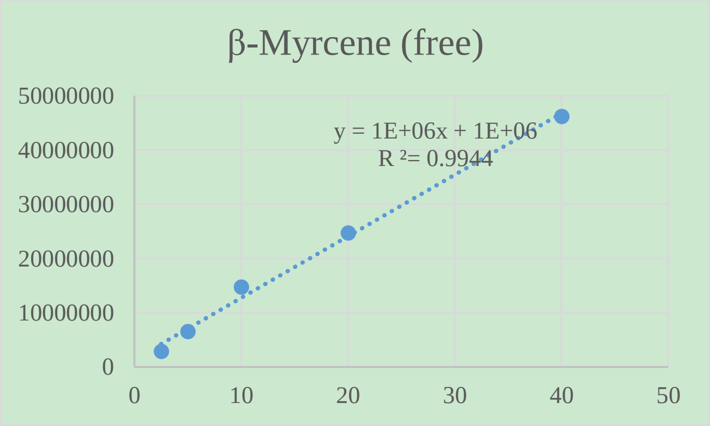
β-Myrcene (free)
y = 1E+06x + 1E+06
R ²= 0.9944
50000000
40000000
30000000
20000000
10000000
0
0
10
20
30
40
50

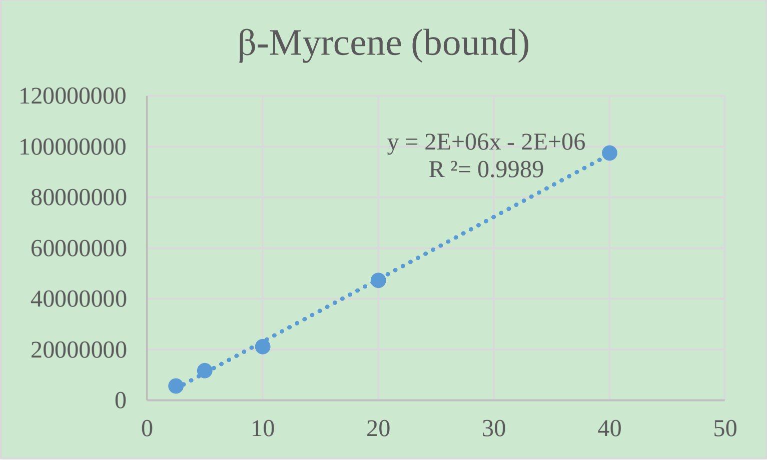
β-Myrcene (bound)
y = 2E+06x - 2E+06
R ²= 0.9989
120000000
100000000
80000000
60000000
40000000
20000000
0
0
10
20
30
40
50

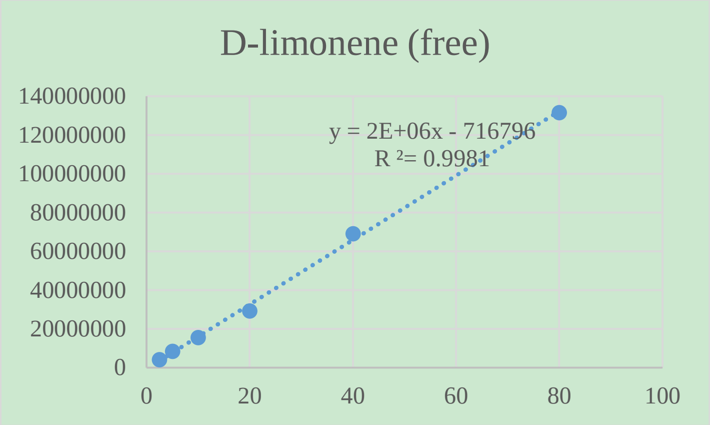
D-limonene (free)
y = 2E+06x - 716796
R ²= 0.9981
140000000
120000000
100000000
80000000
60000000
40000000
20000000
0
0
20
40
60
80
100

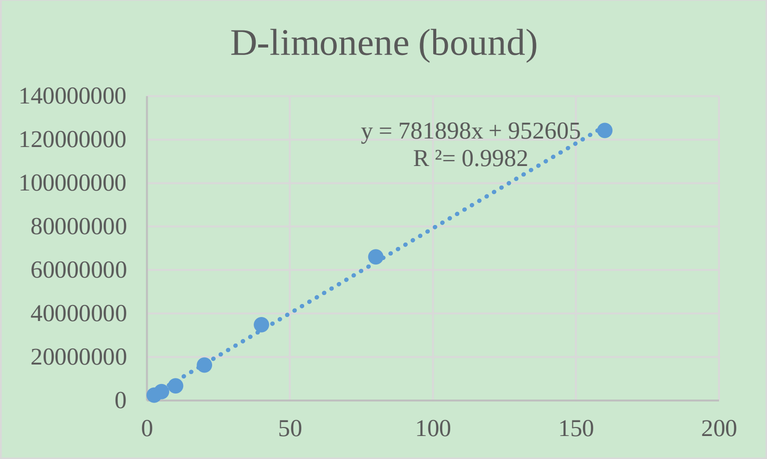
D-limonene (bound)
y = 781898x + 952605
R ²= 0.9982
140000000
120000000
100000000
80000000
60000000
40000000
20000000
0
0
50
100
150
200

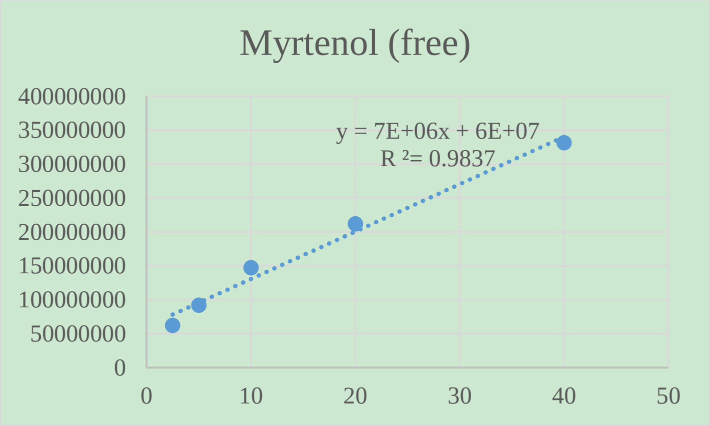
Myrtenol (free)
y = 7E+06x + 6E+07
R ²= 0.9837
400000000
350000000
300000000
250000000
200000000
150000000
100000000
50000000
0
0
10
20
30
40
50

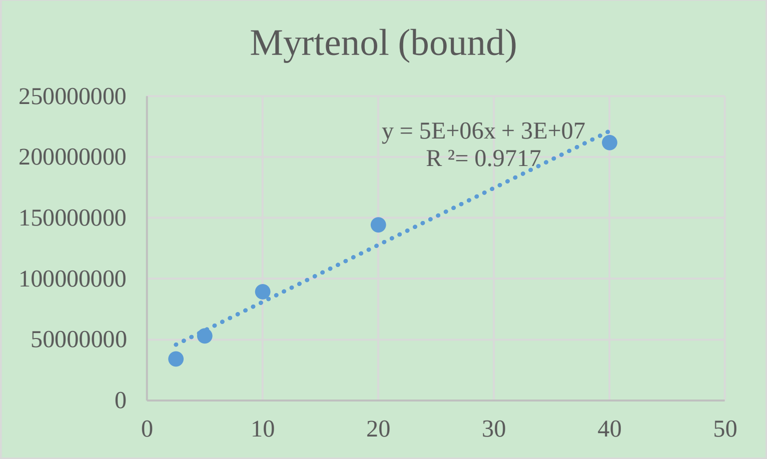
Myrtenol (bound)
y = 5E+06x + 3E+07
R ²= 0.9717
250000000
200000000
150000000
100000000
50000000
0
0
10
20
30
40
50

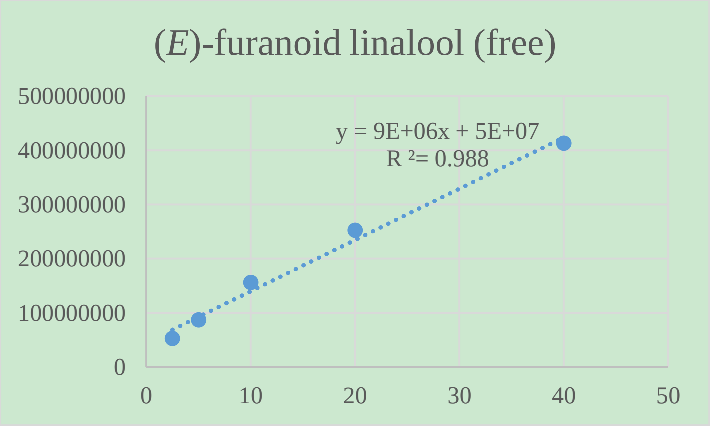
(*E*)-furanoid linalool (free)
y = 9E+06x + 5E+07
R ²= 0.988
500000000
400000000
300000000
200000000
100000000
0
0
10
20
30
40
50

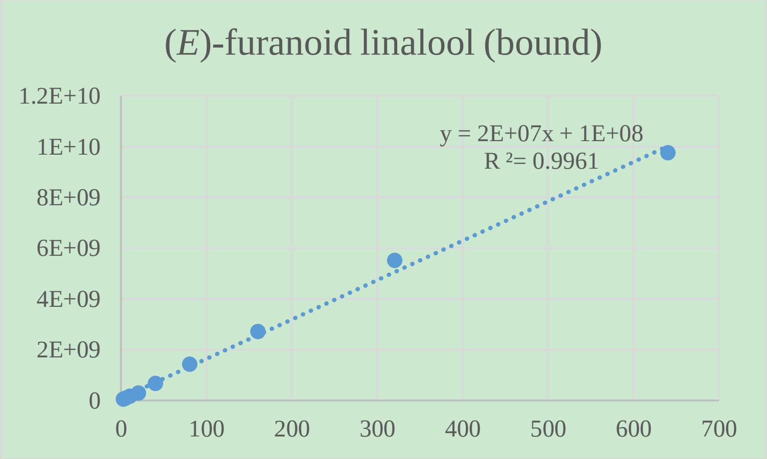
(*E*)-furanoid linalool (bound)
y = 2E+07x + 1E+08
R ²= 0.9961
1.2E+10
1E+10
8E+09
6E+09
4E+09
2E+09
0
0
100
200
300
400
500
600
700

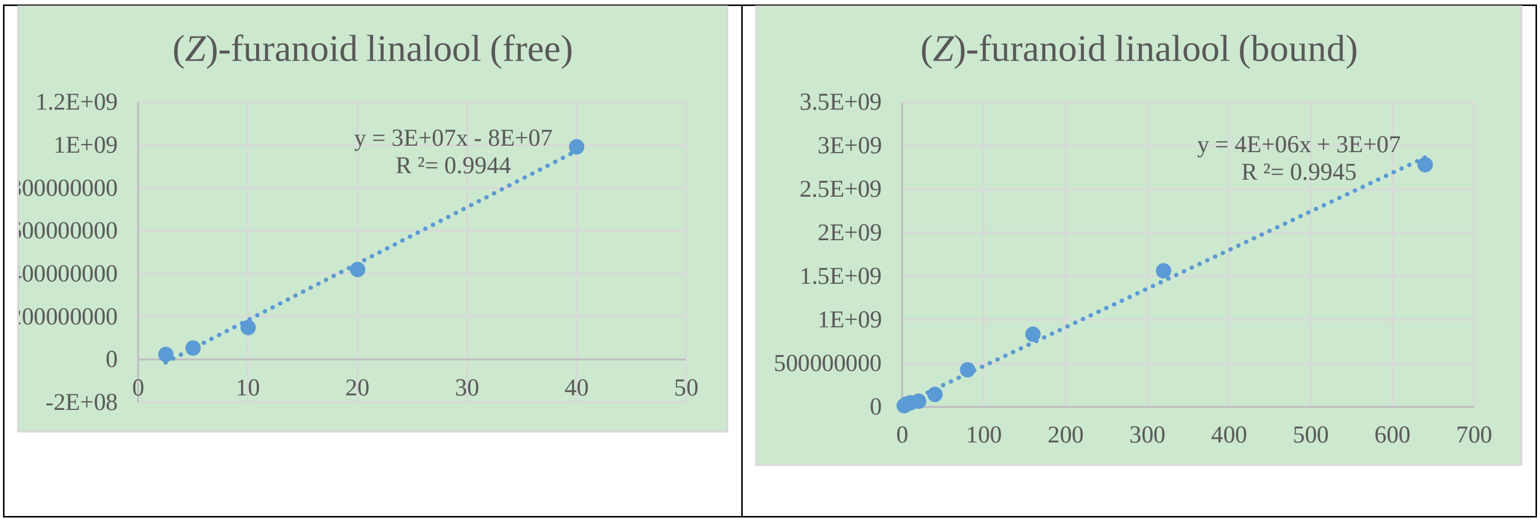

Figure S1. Calibration curves of free compounds (A. pH 3.2, citrate-phosphate buffer solution) and bound volatile compounds (B. pH 5.0, citrate-phosphate buffer solution). The standard solutions were diluted in order to obtain from 5-9 concentration levels. Linear range of free and bound standard were 2.5-640 ng/g.

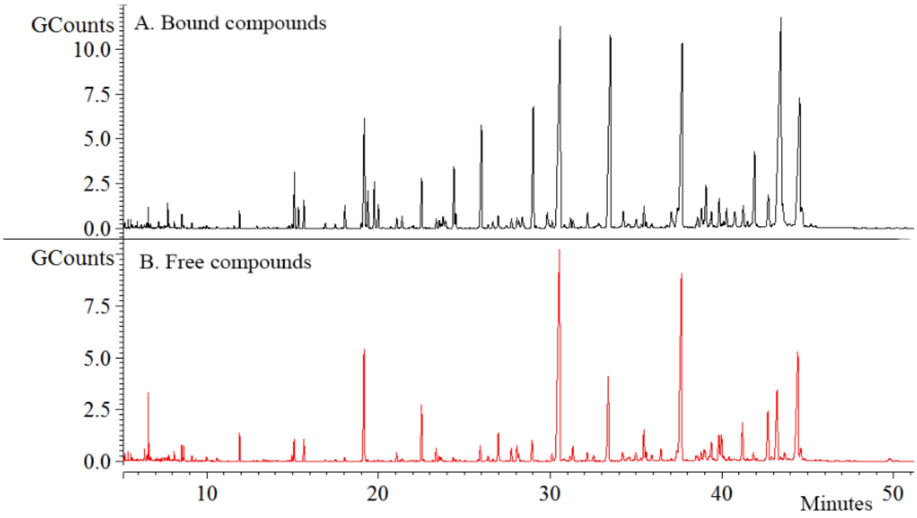

Fig S2 represented chromatogram of GC-MS. A. chromatogram for bound compounds. B. chromatogram for free compounds.

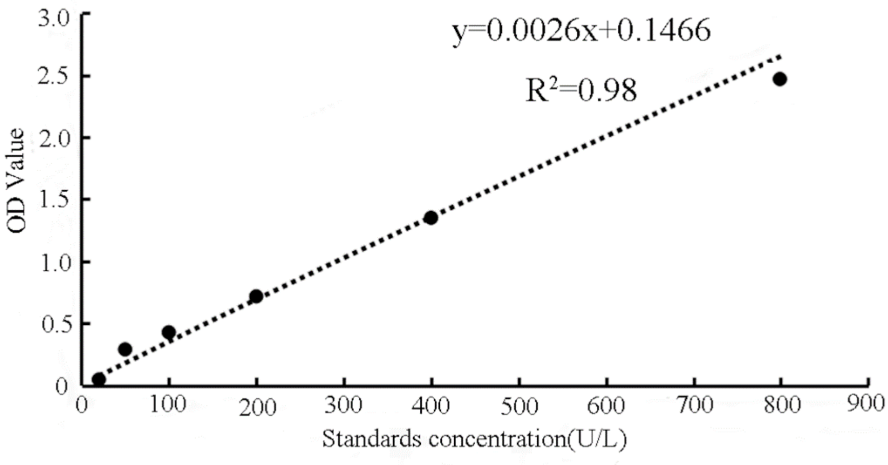

Fig S3 Calibration curves of TPS standards. The standard solutions were diluted in order to obtain from 5 concentration levels. Linear range of free and bound standard were 0-800 U/L.
